# Preliminary study on alterations of altitude road traffic in China from 2006 to 2013

**DOI:** 10.1371/journal.pone.0171090

**Published:** 2017-02-10

**Authors:** Hui Zhao, Zhiyong Yin, Hongyi Xiang, Zhikang Liao, Zhengguo Wang

**Affiliations:** Chongqing Key Laboratory of Vehicle Crash/Bio-Impact and Traffic Safety, Department 4th, Institute of Surgery Research, Daping Hospital, Third Military Medical University, Chongqing, China; Beihang University, CHINA

## Abstract

**Introduction:**

Road traffic can play an important role in strengthening regional economic activities, especially at high altitude, and it is necessary to know important traffic-related information. Although previous studies reported on road traffic in China, there has been little research on high-altitude road traffic to date.

**Method:**

The annual official census of road traffic safety from 2006 to 2013 was used to obtain data on the general population, registered drivers, registered vehicles, newly built roads, road traffic accidents (RTAs), mortality rate per 100 000 populations and per 10 000 vehicles in high-altitude provinces, including Tibet, Qinghai, Xinjiang, Gansu, Yunnan, Sichuan, and Chongqing. These provincial data were reviewed retrospectively, with the national data as the reference. Statistical analysis (i.e., t test) was used to compare the estimated average annual change rate of population, number of registered drivers, registered vehicles, and newly built roads in high-altitude provinces with the national rates.

**Results:**

Compared with the national data, there are significantly higher annual rates of population growth in Tibet and Xinjiang, registered drivers in Gansu, registered vehicles in Gansu, Sichuan, and Chongqing, and newly built roads in Tibet and Qinghai. Among the investigated provinces, Tibet, Qinghai, and Yunnan had a higher proportion of the roads with the high class. RTAs and RTA-induced casualties in the high-altitude provinces indicated a decreasing trend. The mortality rate per 10 000 vehicles and per 100 000 populations showed a decreasing trend, while the RTA-related mortality rate in Tibet, Qinghai, Xinjiang and Gansu remained high.

**Conclusions:**

Major changes for road traffic in high-altitude provinces have occurred over the past decade; however, the RTA-related mortality rate in high-altitude provinces has remained high. This study furthers understanding about road traffic safety in China; further studies on road traffic safety at high altitude should be performed.

## Introduction

Road traffic can play an important role in strengthening regional economic activities, especially at high altitude. With increasing road traffic around the world, road traffic accidents (RTAs) represent a great threat to public health, especially in middle- and low- income countries. According to the World Health Organization [1–2], over 1.2 million people die each year on the world’s roads, and up to 50 million people incur non-fatal injuries as a result of RTAs. The WHO [3] predicted that, by 2030, road traffic deaths will become the fifth leading cause of death if urgent action is not taken in time.

In the past decade, RTAs in China have led to numerous injuries and fatalities, causing a major social burden. The number of RTA-related injuries and fatalities in China in 2012 were up to 213 724 and 58 539, respectively, according to the data issued by the Bureau of Traffic Management of the Ministry of Public Security of the People’s Republic of China [4]. Qiu et al. [5] and Huang et al. [6] suggested that RTA-induced casualties are likely to be severely underestimated in police-issued data.

Developments affecting road traffic elements, namely, drivers, vehicles, and roads differed among regions with different economic levels or environmental conditions [7–8]. High-altitude regions, accounting for a large proportion of China, are situated mostly in the western and southern provinces of China, including Tibet, Qinghai, Xinjiang, Gansu, Yunnan, and Sichuan. However, compared with low-altitude areas, developing roads at high altitudes has been difficult because of the mountainous features of the land and environmental conditions, i.e. sequential storms, constant winds, extreme cold, and hypoxia [7, 9].

Although there have been numerous studies about road traffic in China recently [5, 10–13], few studies on high-altitude road traffic in China have been published [7]. Adverse weather and road conditions have the potential to affect the likelihood or motor vehicle fatalities through several pathways [14]. In previously reported studies concerning high-altitude road traffic, the injuries or fatalities were investigated in local region [8, 11, 15–16], in which austere environmental conditions and rugged mountainous roads were considered the main causes contributing to the high incidence of disastrous outcomes associated with RTAs at high altitude.

It is therefore necessary to develop a more comprehensive understanding of the characteristics of road traffic at high altitude, such as changes in road traffic elements, prior to developing prevention measures for high-altitude RTAs using multidisciplinary approach [7, 15, 17–18]. The purpose of this study, therefore, is to retrospectively analyze recently reported official data, and to determine the characterization of road traffic at high altitude.

## Methods

**Fig 1:**
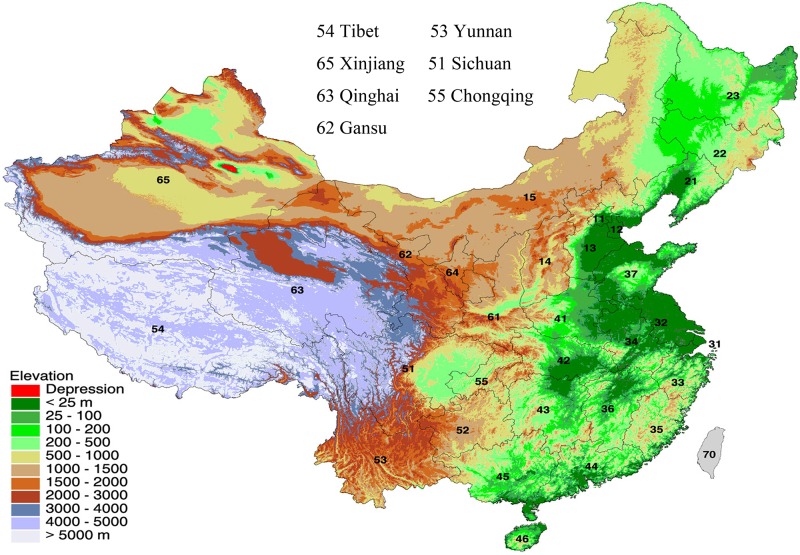
Topographic map of Mainland China. The compiled data were used to evaluate the changes in road traffic, the annual change rate of the population, as well as the number of registered vehicles and drivers, newly built roads. The average annual change rates were determined and the data in high altitude provinces were compared with the national values via statistical analysis, (t test), using SPSS^®^ software. A p<0.05 was considered statistically significant.

A research team was established and trained to collect road traffic-pertinent data. We compiled the annual census data from 2006 to 2013 issued by the Bureau of Traffic Management of the Ministry of Public Security of the People’s Republic of China [4, 19–25], in which the traffic-pertinent elements such as populations, vehicles, drivers, roads, accidents, injuries and fatalities, were officially reported. We then examined the population, registered vehicles and drivers, newly built roads, road types, RTAs, RTA-induced injuries and fatalities, and the mortality rate per 10 000 vehicles and per 100 000 populations in high-altitude provinces of China. The investigated provinces were Tibet, Qinghai, Xinjiang, Gansu, Yunnan, Sichuan, and Chongqing (Fig 1), in which the elevations of the investigated provinces were indicated using the various colors. National data were used as the reference point. The investigated traffic-pertinent elements (S1–S8 Tables) were uploaded as the supporting files by the altitude provinces and national.

## Results

In contrast to the annual population growth between 2006 and 2013 (Table 1), a sharp increase in the number of the registered drivers in high-altitude provinces was found (Table 2). Gansu had a significantly higher average annual growth rate in the number of registered drivers (0.13±0.030), compared with the national rate (0.093±0.018; p<0.05).

**Table 1:** Population from 2006 to 2013 in high-altitude provinces and nationally in China.

		2006	2007	2008	2009	2010	2011	2012	2013
Tibet	Num(1,000,000)	2.77	2.81	2.84	2.87	2.90	3.01	3.03	3.12
	Increase(%)	--	1.44	1.07	1.06	1.05	3.80	0.07	2.97
Qinghai	Num(1,000,000)	5.43	5.47	5.52	5.54	5.57	5.63	5.68	5.58
	Increase(%)	--	0.74	0.91	0.36	0.54	1.08	0.89	-1.76
Xinjiang	Num(1,000,000)	20.10	20.50	20.95	21.31	21.59	21.85	22.09	22.64
	Increase(%)	--	1.99	2.20	1.72	1.31	1.20	1.10	2.49
Gansu	Num(1,000,000)	25.94	26.06	26.17	26.28	26.35	25.60	25.64	25.82
	Increase(%)	--	0.46	0.42	0.42	0.27	-2.85	0.16	0.70
Yunnan	Num(1,000,000)	44.50	44.83	45.14	45.43	45.71	46.02	46.31	48.69
	Increase(%)	--	0.74	0.69	0.64	0.61	0.68	0.63	5.14
Sichuan	Num(1,000,000)	87.50	81.69	81.27	81.38	81.85	80.54	80.50	81.07
	Increase(%)	--	-6.64	-0.51	0.14	0.58	-1.60	-0.05	0.71
Chongqing	Num(1,000,000)	27.98	28.08	28.16	28.39	28.59	28.85	29.19	29.70
	Increase(%)	--	0.36	0.28	0.82	0.71	0.91	1.18	1.74
National	Num(1,000,000)	1307.56	1314.48	1321.29	1328.02	1334.74	1341.00	1347.35	1360.72
	Increase(%)	--	0.53	0.52	0.51	0.51	0.47	0.47	0.99

**Table 2:** Number of registered drivers from 2006 to 2013 in high-altitude provinces and nationally in China.

		2006	2007	2008	2009	2010	2011	2012	2013
Tibet	Num(1,000,000)	0.15	0.15	0.19	0.21	0.19	0.21	0.22	0.24
	Increase(%)	--	0.00	26.67	10.53	-9.52	10.53	4.76	9.09
Qinghai	Num(1,000,000)	0.47	0.50	0.66	0.73	0.79	0.88	0.97	1.03
	Increase(%)	--	6.38	32.0	10.60	8.22	11.39	10.23	6.19
Xinjiang	Num(1,000,000)	2.21	2.22	2.69	2.91	3.15	3.47	3.85	4.17
	Increase(%)	--	4.53	21.17	8.18	8.24	10.16	10.95	8.31
Gansu	Num(1,000,000)	1.44	1.56	1.76	2.01	2.27	2.64	3.06	3.39
	Increase(%)	--	8.33	12.82	14.20	12.93	13.60	15.91	10.78
Yunnan	Num(1,000,000)	4.17	4.80	5.65	6.35	7.00	7.81	8.69	9.43
	Increase(%)	--	15.11	17.71	12.39	10.24	11.57	11.27	8.52
Sichuan	Num(1,000,000)	6.77	7.60	8.25	10.32	11.48	12.65	13.86	14.91
	Increase(%)	--	12.26	8.55	25.09	11.24	10.19	9.57	7.58
Chongqing	Num(1,000,000)	1.84	2.09	2.34	2.67	3.22	4.08	4.74	5.22
	Increase(%)	--	0.14	0.11	0.15	0.21	0.27	0.16	0.10
National	Num(1,000,000)	150.12	163.89	180.66	199.77	212.93	235.62	261.22	279.12
	Increase(%)	--	9.17	10.23	10.58	6.59	10.66	10.87	6.85

The number of registered vehicles in China has increased by 172%, from 145.23 million in 2006 to 250.14 million in 2013(Table 3). The average annual growth rate in the number of registered vehicles in Gansu, Sichuan and Chongqing, was 0.14±0.042, 0.16±0.059, and 0.18±0.11, respectively, significantly higher than the national rate of 0.13±0.030 (p<0.05).

**Table 3:** Number of registered vehicles from 2006 to 2013 in high-altitude provinces and nationally in China.

		2006	2007	2008	2009	2010	2011	2012	2013
Tibet	Num(1,000,000)	--	0.13	0.15	0.20	0.23	0.26	0.27	0.31
	Increase(%)	--	--	15.78	32.85	12.22	13.84	3.87	17.17
Qinghai	Num(1,000,000)	0.41	0.43	0.48	0.53	0.60	0.69	0.78	0.85
	Increase(%)	--	6.45	10.23	11.23	13.81	14.37	12.71	9.29
Xinjiang	Num(1,000,000)	2.17	2.36	2.50	2.74	3.07	3.49	3.93	4.33
	Increase(%)	--	8.66	5.90	9.67	12.26	13.48	12.57	10.27
Gansu	Num(1,000,000)	1.26	1.36	1.50	1.78	2.12	2.45	2.78	3.08
	Increase(%)	--	8.22	10.47	18.44	19.29	15.57	13.46	10.65
Yunnan	Num(1,000,000)	3.68	4.66	5.25	6.17	7.24	8.37	9.39	10.08
	Increase(%)	--	26.35	12.77	17.55	17.31	15.56	12.22	7.31
Sichuan	Num(1,000,000)	5.35	5.94	6.91	8.36	9.59	10.64	11.66	12.37
	Increase(%)	--	11.11	16.40	20.91	14.70	10.99	9.53	6.09
Chongqing	Num(1,000,000)	1.33	1.43	1.62	2.01	2.71	3.39	3.91	4.09
	Increase(%)	--	7.92	12.53	24.04	34.85	25.30	15.30	4.59
National	Num(1,000,000)	145.23	159.78	169.89	186.58	207.06	224.79	239.89	250.14
	Increase(%)	--	10.02	6.33	9.83	10.98	8.56	6.71	4.27

**Fig 2:**
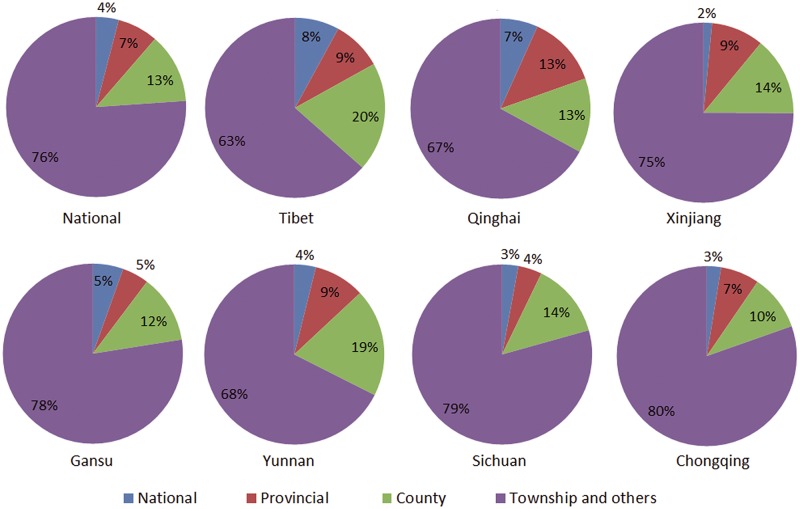
Proportions of road types among high-altitude provinces in 2013.

The number of newly built roads in high-altitude provinces has increased considerably since 2006 (Table 4), and the average annual growth rates of newly built roads in Tibet (0.065±0.036) and Qinghai (0.056±0.027) were significantly higher than the national rate (0.033±0.0058; p<0.05). In 2013, the proportion of roads with the high class was distinctly higher in Tibet, Qinghai, and Yunnan compared with the national rate (Fig 2).

**Table 4:** Newly built roads from 2006 to 2013 in high-altitude provinces and nationally in China (Km).

		2006	2007	2008	2009	2010	2011	2012	2013
Tibet	Num(10,000)	4.48	4.86	5.13	5.38	6.08	6.31	6.52	7.06
	Increase(%)	--	8.48	5.56	4.93	12.94	3.78	3.31	8.27
Qinghai	Num(10,000)	4.77	5.26	5.66	6.01	6.22	6.43	6.60	7.01
	Increase(%)	--	10.27	7.63	6.17	3.41	3.37	2.66	6.26
Xinjiang	Num(10,000)	14.37	14.52	14.67	15.07	15.28	15.52	16.59	17.02
	Increase(%)	--	1.03	0.99	2.74	1.43	1.51	6.93	2.56
Gansu	Num(10,000)	8.56	10.06	10.56	11.40	11.89	12.37	13.12	13.36
	Increase(%)	--	5.20	5.0	7.92	4.28	4.05	6.07	1.83
Yunnan	Num(10,000)	19.85	20.03	20.38	20.60	20.92	21.45	21.91	22.94
	Increase(%)	--	0.93	1.71	1.12	1.55	2.53	2.11	1.77
Sichuan	Num(10,000)	16.47	18.94	22.45	24.92	26.61	28.33	29.35	30.18
	Increase(%)	--	15.0	18.53	11.00	6.78	6.46	3.61	2.83
Chongqing	Num(10,000)	10.03	10.47	10.86	11.10	11.69	11.86	12.07	12.28
	Increase(%)	--	4.39	3.75	2.12	5.41	1.38	1.83	1.75
National	Num(10,000)	345.70	358.37	373.02	386.08	400.82	410.64	423.75	435.62
	Increase(%)	--	3.67	4.09	3.50	3.82	2.45	3.19	2.80

Although there are fluctuations in the rate of RTAs and RTA-induced casualties in Tibet and Qinghai (Tables 5 and 6), the data indicate an overall decreasing trend in the investigated provinces.

**Table 5:** Road traffic accidents from 2006 to 2013 in the high altitude provinces and nationally in China.

		2006	2007	2008	2009	2010	2011	2012	2013
Tibet	Num	751	638	600	678	781	943	725	717
	Increase(%)	--	-15.05	-5.96	13.0	15.19	20.74	-23.12	-1.10
Qinghai	Num	939	1041	1120	1146	1206	1163	1096	1065
	Increase(%)	--	10.86	7.59	2.32	5.23	-3.57	-5.75	-2.83
Xinjiang	Num	7428	6735	5684	5120	5298	5182	4943	4944
	Increase(%)	--	-9.33	-15.61	-9.92	3.48	2.19	4.61	0.02
Gansu	Num	4828	3808	3364	2937	3090	3027	2954	2915
	Increase(%)	--	-21.13	-11.66	-12.69	5.21	2.04	2.41	1.32
Yunnan	Num	6692	5425	5037	5075	4739	5022	3941	3748
	Increase(%)	--	-18.93	-7.15	0.75	-6.62	5.97	-21.53	-4.90
Sichuan	Num	24338	21598	17037	21680	13072	11860	10024	9571
	Increase(%)	--	-11.26	-21.12	27.25	-39.70	-9.27	-15.48	-4.51
Chongqing	Num	9001	8236	7262	5992	5908	5729	5791	5642
	Increase(%)	--	-8.50	-11.83	-17.49	-1.40	-3.03	1.08	-2.57
National	Num	378781	327209	265204	238351	219521	210812	204196	198394
	Increase(%)	--	-13.62	-18.95	-10.13	-7.90	-3.97	-3.14	-2.84

**Table 6:** Casualties caused by the accidents from 2006 to 2013 in high-altitude provinces and nationally in China.

		2006	2007	2008	2009	2010	2011	2012	2013
Tibet	Num	1573	1179	1044	1117	1378	1330	1144	1171
	Increase(%)	--	-23.29	-11.45	6.99	23.37	-3.48	-13.99	2.36
Qinghai	Num	1861	1741	1895	1912	2067	1979	1946	1765
	Increase(%)	--	-6.45	8.85	0.90	8.11	-4.26	-1.67	-9.30
Xinjiang	Num	11456	10105	8614	7867	8051	7888	7406	7576
	Increase(%)	--	-11.79	-14.76	-8.67	2.33	-2.03	-6.11	2.30
Gansu	Num	7016	5834	5247	4886	5234	5083	4783	4771
	Increase(%)	--	-16.85	-10.06	-6.88	7.12	-2.89	-5.90	-0.25
Yunnan	Num	10959	8572	7999	8437	7786	8273	6803	6320
	Increase(%)	--	-21.78	-6.69	5.47	-7.72	6.25	-17.77	-7.10
Sichuan	Num	28428	25388	20381	24737	16003	14665	12732	12229
	Increase(%)	--	-10.69	-19.72	21.37	-35.31	-8.36	-13.18	-3.95
Chongqing	Num	13381	13111	11953	10209	9475	9513	9528	8853
	Increase(%)	--	-2.02	-8.83	-14.59	-7.19	0.40	0.16	-7.08
National	Num	520594	462091	378403	342884	319300	299808	284324	272263
	Increase(%)	--	-11.24	-18.11	-9.39	-6.88	-6.11	-5.17	-4.24

**Fig 3:**
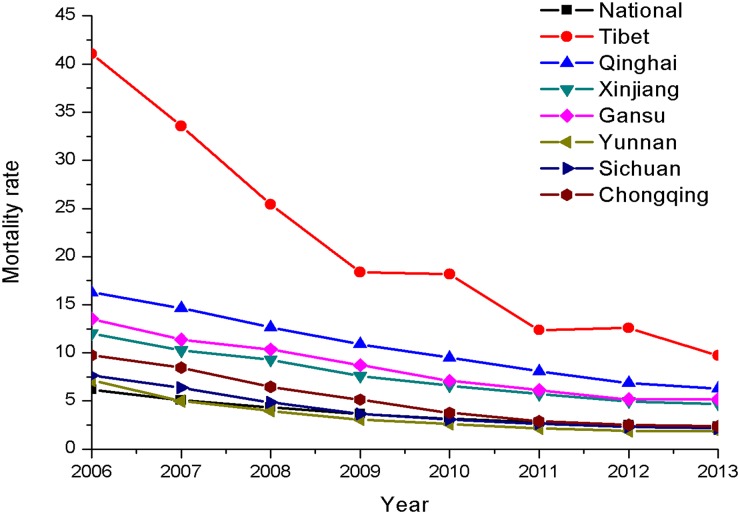
Mortality rate per 10 000 vehicles from 2006 to 2013.

**Fig 4:**
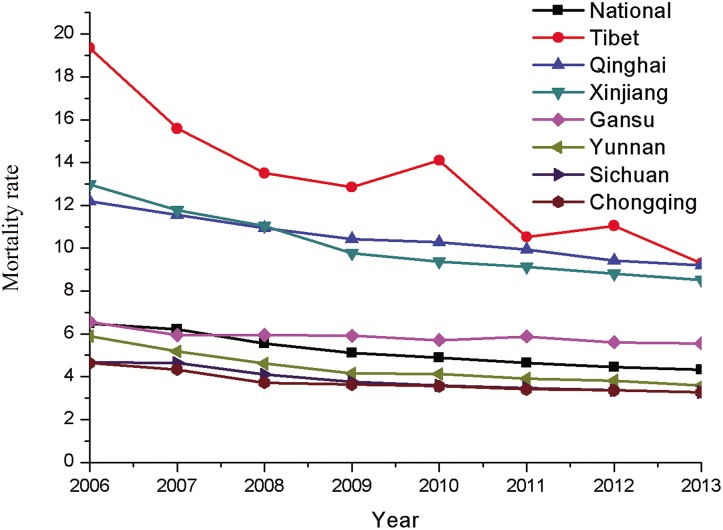
Mortality rate per 100 000 populations from 2006 to 2013.

There has been a general decrease in the mortality rate per 10 000 vehicles and per 100 000 populations in high-altitude provinces since 2006; however, the mortality rate remained remarkably high in Tibet, Qinghai, Xinjiang, and Gansu (Figs 3 and 4). In 2013, the mortality rate per 10 000 vehicles in Tibet was the highest among the studied provinces at 9.7, followed by Qinghai at 6.3. The rate in China was 1.85. The mortality rate per 100 000 populations was the highest in Tibet at 9.3, followed by Qinghai at 9.2, and the rate was 4.32 in China.

## Discussion

Developing road traffic in high-altitude is important for improving the economic activities in such regions, and remains a considerable challenge due to environmental conditions. Currently, there remains a paucity of studies regarding the road traffic in high-altitude areas. In the current study, road traffic in high-altitude provinces was studied in great detail using official census data from 2006 to 2013. Road traffic-pertinent information related to drivers, vehicles, and roads, was analyzed retrospectively and compared with the national data. The authors considered that the presently reported results with regard to road traffic safety at high altitude may be valuable for attracting the attention of experts, including those engaged in the research on safe roads, safe vehicles and safe people to reduce the number of injuries and fatalities [18], especially for low or middle income countries [17].

The current study indicates that distinct changes have occurred in road traffic in high-altitude provinces in recent years. In our study there is a significantly higher average annual growth rate in populations of Tibet and Xinjiang, registered drivers in Gansu, registered vehicles in Gansu, Sichuan, and Chongqing, newly built roads in Tibet and Qinghai, compared with the national rate. During the same period, the annual growth rate of the population, and registered vehicles in Nepal, close to Tibet, China, also showed a dramatic increase [1, 3]. For high-income countries, such as United States, Germany, and France, however, there is a slight increase in registered vehicles [26].

A difficulty remained in building the roads in high-altitude areas due to environmental conditions. Moreover, the roads at high altitude were generally considered poor owing to the topographic features, while the roads were developed remarkably in high-altitude provinces recently. In 2013 the roads with a high class in high-altitude provinces accounted for a high proportion of all roads in comparison to the national average (Fig 2).

RTAs and RTA-induced casualties in high-altitude provinces showed a decreasing trend, while some fluctuations were observed in Qinghai and Tibet, as shown in Tables 5 and 6. Brude and Elvik [27] analyzed the fatalities induced by RTAs from six countries and then concluded that road safety policy is more effective to influence the changes of the number of traffic fatalities. The authors agreed that there exists some potential factors to affect the likelihood of RTAs-induced fatalities, such as weather and road conditions suggested by Saha et al. [14], while did not concluded the reason for the fluctuation at high-altitude from the reported official census data in the study. Mishra et al. [15] found that an increased prevalence of RTA occurred at the beginning (i.e., 55%) and end (i.e., 19%) of a journey. Analyzing RTAs at high altitude, Zhu et al. [16] concluded that human error led to accidents more frequently. By studying a total of 1894 RTA-related injuries in Tibet and Qinghai, Wang et al. [11] found that the residents of low-altitude areas who had acute exposure to high altitudes accounted for 61% of the injured, and inferred that hypoxia at high altitude, unclear road signs, and driving fatigue mainly contributed to the high incidence at high altitude. Because of a lack of detailed data, further analysis has not been done in the current study.

There are a huge discrepancy in mortality rate per 10 000 vehicles and per 100 000 populations worldwide. The mortality rate per 10 000 vehicles and per 100 000 populations was 1.2 and 10.6 in USA, 0.8 and 5.1 in France, and 0.6 and 4.3 in Germany, respectively [2, 26]. As compared to US and EU countries, it was found the mortality rate per 10 000 vehicles and per 100 000 populations are remarkably high in China according to the data shown in Figs 3 and 4. Although the mortality rate showed a decreasing trend, the mortality rate for traffic injury at high altitude remained high in comparison to the national rate. In the study, the mortality rate of 10 000 vehicles in Tibet and Qinghai in 2013 was over 5 and 3 times the national rate, while the mortality rate per 100 000 populations in Tibet and Qinghai was about twice the national rate. In Nepal 962 people died in RTAs in 2007, 1689 died in 2010, with an estimated mortality rate of 6.4 individuals per 100 000 populations [1, 3]. Mishra et al. [15] concluded that RTA-related fatalities in Nepal could be prevented if the injured patients could be transported to hospital in a timely manner.

In addition to the high incidence of RTAs in the high-altitude provinces, a lack of valid and timely rescue service may contribute to the high mortality rate at high altitude, especially in Tibet and Qinghai. Wang et al. [28] concluded that there are no standard procedures for the rescue stage before hospital admission and poorly trained emergency medical service workers and insufficient onsite care result in a low success rate of pre-hospital resuscitation in China. In addition, there are few health care facilities along national roads, especially those connecting Qinghai and Tibet, making it difficult for rescue workers to arrive in time [11]. In the study, only about 2.5% of road traffic injuries received appropriate pre-hospital treatment, and fatalities accounted for 5.7% of the inpatients sustaining traffic crashes. Furthermore, hypoxia and extreme cold may further harm RTA-injured patients, resulting in disastrous outcomes, shown in the previously reported studies [9, 29–31]. For injuries in high-altitude provinces, compared with in lowland areas, significant discrepancies were found in the demographics, hospital stay and surgery of inpatients [32]. More attention should be paid to high-altitude traumatic patients [32–33].

This study has some shortcomings. For example, the data from police department may lack the detailed information that may be useful for making measurements to reduce the incidence at high altitude, including road users, accidental time, accident fields, etc. The annual census rates of RTAs and the casualties of RTAs at high altitude may be underestimated severely owing to the police data source [5–6]. In addition, casualties in the police records were defined as having died within 7 days of an RTA; however, casualties are those who die with 30 or 35 days after an RTA in other countries. The hospital data associated with road traffic injury at high altitude were not used in the current study. Such data may be important in preventing against injury and death. Using hospital data, rescue workers and doctors may learn the characteristics of traffic injuries at high altitude and train more effectively to treat such injuries. They may also be important for experts engaged in vehicle safety research, who could use it to improve vehicle safety [7, 15]. Finally, owing to the limited accident information from the census, the data were not further analyzed.

## Conclusion

The annual census data on road traffic issued by the Bureau of Traffic Management of the Ministry of Public Security of the People’s Republic of China from 2006 to 2013 were reviewed retrospectively. Our results suggest that significant changes have occurred in road traffic-related factors affecting drivers, vehicles, and roads. Compared with the national data, a significantly higher average annual growth rate was observed in the populations of Tibet and Xinjiang, as well as in registered drivers in Gansu, registered vehicles in Sichuan, Gansu, and Chongqing, and newly-built roads in Tibet and Qinghai. In 2013, there was a high proportion of roads with a high class in Tibet, Qinghai, and Yunnan. The overall RTA-related mortality rate showed a decreasing trend from 2006 to 2013; however the mortality rate remained high in Tibet, Qinghai, Xinjiang, and Gansu, compared with the national rate. Despite the limitations of this study, the findings may attract more attention to road traffic safety at high altitude, and encourage further studies on road traffic in high-altitude settings.

## Supporting information

S1 Table Road traffic-pertinent elements in Tibet from 2006 to 2013. The information was compiled from annual census data issued by the Bureau of Traffic Management of the Ministry of Public Security of the People’s Republic of China.
(XLS)

S2 Table Road traffic-pertinent elements in Qinghai from 2006 to 2013. The information was compiled from annual census data issued by the Bureau of Traffic Management of the Ministry of Public Security of the People’s Republic of China.
(XLS)

S3 Table Road traffic-pertinent elements in Xinjiang from 2006 to 2013. The information was compiled from annual census data issued by the Bureau of Traffic Management of the Ministry of Public Security of the People’s Republic of China.
(XLS)

S4 Table Road traffic-pertinent elements in Gansu from 2006 to 2013. The information was compiled from annual census data issued by the Bureau of Traffic Management of the Ministry of Public Security of the People’s Republic of China.
(XLS)

S5 Table Road traffic-pertinent elements in Yunnan from 2006 to 2013. The information was compiled from annual census data issued by the Bureau of Traffic Management of the Ministry of Public Security of the People’s Republic of China.
(XLS)

S6 Table Road traffic-pertinent elements in Sichuan from 2006 to 2013. The information was compiled from annual census data issued by the Bureau of Traffic Management of the Ministry of Public Security of the People’s Republic of China.
(XLS)

S7 Table Road traffic-pertinent elements in Chongqing from 2006 to 2013. The information was compiled from annual census data issued by the Bureau of Traffic Management of the Ministry of Public Security of the People’s Republic of China.
(XLS)

S8 Table National road traffic-pertinent elements from 2006 to 2013. The information was compiled from annual census data issued by the Bureau of Traffic Management of the Ministry of Public Security of the People’s Republic of China.
(XLS)
